# Classification in Networked Data with Heterophily

**DOI:** 10.1155/2013/236769

**Published:** 2013-04-30

**Authors:** Zhenwen Wang, Fengjing Yin, Wentang Tan, Weidong Xiao

**Affiliations:** College of Information System and Management, National University of Defense Technology, Changsha 410073, China

## Abstract

In the real world, a large amount of data can be described by networks using relations between data. The data described by networks can be called networked data. Classification is one of the main tasks in analyzing networked data. Most of the previous methods find the class of the unlabeled node using the classes of its neighbor nodes. However, in the networks with heterophily, most of connected nodes belong to different classes. It is hard to get the correct class using the classes of neighbor nodes, so the previous methods have a low level of performance in the networks with heterophily. In this paper, a probabilistic method is proposed to address this problem. Firstly, the class propagating distribution of the node is proposed to describe the probabilities that its neighbor nodes belong to each class. After that, the class propagating distributions of neighbor nodes are used to calculate the class of the unlabeled node. At last, a classification algorithm based on class propagating distribution is presented in the form of matrix operations. In empirical study, we apply the proposed algorithm to the real-world datasets, compared with some other algorithms. The experimental results show that the proposed algorithm performs better when the networks are of heterophily.

## 1. Introduction

Classification is one of the main tasks in the data mining field. Most traditional classification methods assume the data instances are independent and assign class labels to the data instances using their attribute values. Besides the attribute information, the connections between data instances can be observed. These connections can be used to classify the data instances. For example, in the field of social network analyzing, it is requested to infer the missing community information of individuals using the interactions between them and other individuals whose community information is observed. This problem can be taken as classification in networked data. Networked data is a name for a group of data that can be described as networks where nodes represent the data instances and edges the connections between them. Classification in networked data is to predict the classes of unlabeled nodes based on the network and the classes of labeled nodes [1]. 

Many methods have been developed for classification in networked data, including collective inference [2–5] and random walk on graphs [6–8]. These methods predict classes of unlabeled nodes based on the classes of their neighbor nodes. In fact, they are the homophily-based methods. The phenomenon of homophily, nodes with similar characters having tendency to interconnect with each other, exists in many real-world networks [9]. Therefore, these methods can return the reasonable results in the networks with high homophily degree. However, there are many heterophilous networks, in which the homophily degrees are low and most of connected nodes have the different class labels. Consequently, those homophily-based methods, which use the classes of neighbor nodes to predict classes of nodes, cease to be effective.

A probabilistic method for classification in networked data is presented in this study. This method calculates the class of the unlabeled nodes based on a probabilistic approach. The main idea is that the class of the unlabeled node is influenced by their neighbor nodes and the influence from a node is measured by the probabilities that its neighbor nodes belong to each class. A classification algorithm is proposed based on this idea. In empirical study, we compare the proposed algorithm with three classification algorithms on six real-world networks. The experimental results show that the proposed algorithm provides better performance on the networks with heterophily.

## 2. Related Works

The classification problem in networked data is studied in this work. *G* = 〈*V*, *E*〉 represents a network, where *V* is the set of nodes and *E* is the set of edges. This problem can be described as finding the categories of those unlabeled nodes given *G* and the categories of labeled nodes. The research works related to this problem include collective inference, random walk on graphs, and the methods based on the feature extraction. 

Collective inference is a group of methods that are based on a Markov assumption:

(1) $$\begin{array}{c} p\left(y_{i}\;|\;G\right)=p\left(y_{i}\;|\;{𝒩}_{i}\right), \end{array}$$

where *y*_*i*_ is the category of node *v*_*i*_ and *𝒩*_*i*_ is the neighbor of node *v*_*i*_. Many collective inference methods have been developed based on local classifiers including Bayesian classifier [2], normalized Logistic regression [3], maximum entropy model [4], and relational probability trees [5]. These methods based on classifiers have to train a classifier before classifying nodes. Weighted-vote relational neighbor classifier (wvRN) is a simple collective inference method, which does not require the training process and directly computes the categories of unlabeled nodes in the manner of iteration.

The MultiRankWalk (MRW) utilizes random walk on graphs to compute the categories of unlabeled nodes [6]. MRW still uses the neighbor nodes to compute the categories of unlabeled nodes. In MRW, the weighted transition matrix is calculated based on the adjacency matrix and the categories of unlabeled nodes are computed by

(2) $$\begin{array}{c} S^{t+1}=\left(1-d\right)\cdot u+d\cdot Y\cdot S^{t}. \end{array}$$

The matrix **Y** is the weighted transition matrix. The matrix **u** is the initial matrix of node category. The matrix **S** ^**t**^ is the matrix representing the probabilities that each node belongs to each category. *d* is a constant. The method in [7] computes the classes of unlabeled nodes by utilizing a random walk on symmetric normalized Laplacian matrix. [8] considers the probabilities of classes during a random walk. 

Collective inference and random walk on graphs both calculate the class labels of unlabeled nodes based on their neighbor nodes. These methods can be viewed as the group of homophily-based methods. Homophily is the phenomenon revealed in the studies of social networks [10]. Nodes connected by an edge have great possibility to possess the same classes, according to homophily. These methods can realize accurate classification on the networks with high homophily degree. However, in the networks with heterophily, most connected nodes have different class labels, and previous methods encounter accuracy decline. To overcome this problem, Tang and Liu propose SocioDim method, which trains an SVM classifier based on the latent node attributes extracted from the topology of networks [9]. However, the latent node attributes, which are obtained by paying great effort, may not reflect the real character of nodes. It makes it hard for SocioDim to ensure that nodes are classified into correct classes. In this study, a simple method is proposed to classification in the networks with heterophily by introducing a probabilistic approach. 

## 3. A Method Based on Class Propagating Distributions

In the networks with heterophily, most of connected nodes have different classes. In this case, those classification methods based on the classes of the neighbor nodes lose their effectiveness. In this study, the scope on which we focus is expanded to the neighbors of their neighbor nodes and a probabilistic approach is utilized to calculate the class labels of unlabeled nodes. Let *P*_*ic*_ denote the probability that the node *v*_*i*_ has the class *L*_*c*_. The vector **P**_**i**_ = {*P*_*ic*_}_*c*=1_^*M*^ is the class distribution of *v*_*i*_ where *M* is the number of classes. ∑_*c*=1_^*M*^ *P*_*ic*_ = 1. The probabilities {*P*_*ic*_}_*c*=1_^*M*^ can represent the class of the node *v*_*i*_. In the network *G*, some nodes are labeled while others are unlabeled. For the labeled nodes, the class distribution is the vector in which only one element is 1 and the rest are 0. Calculating the classes of unlabeled nodes is to calculate the class distributions of unlabeled nodes. 

The class of a node can be influenced by its neighbor nodes, but the influence is not determined by the classes of its neighbor nodes in this approach. Assume that the node *v*_*i*_ and *v*_*j*_ are connected by an edge. When calculating the class of the nodes *v*_*i*_, the influence *I*_*j*_ from *v*_*j*_ is determined by the classes of the neighbor nodes of *v*_*j*_. If the number of nodes labeled with *L*_*c*_ is larger in the neighbor nodes of the node *v*_*j*_, *I*_*j*_ will make *v*_*i*_ labeled with the class *L*_*c*_ with greater probability. Consider a network *G* with *N* nodes. Let **W** denote the adjacency matrix of *G* and **δ**_**i**_ the influence from the neighbor nodes of *v*_*i*_. **δ**_**i**_ = {*I*_*j*_}_*j*=1,*W*_*ij*_=1_^*N*^. This approach calculates the label of the unlabeled node *v*_*i*_ based on the following assumption:

(3) $$\begin{array}{c} P_{ic}=p\left(y_{i}=c\;|\;\delta_{i}\right). \end{array}$$

According to this assumption, the class distributions of unlabeled nodes are calculated based on {*I*_*j*_}_*j*=1_^*N*^. To measure the influence *I*_*j*_ quantitatively, the class propagating distribution is proposed here. Let *q*_*jc*_ denote the fraction of the nodes that have the class *L*_*c*_ in the neighbor nodes of *v*_*j*_. The vector **q**_**j**_ = [*q*_*j*1_,…, *q*_*jc*_,…, *q*_*jM*_] is called the class propagating distribution of *v*_*j*_. *q*_*jc*_ can be calculated by

(4) $$\begin{array}{c} q_{jc}=\frac{\sum_{i=1}^{N}P_{ic}W_{ij}}{\sum_{k=1}^{N}W_{jk}}. \end{array}$$

The vector **q**_**j**_ is used to measure the influence *I*_*j*_, where the *c*'th element of **q**_**j**_ is larger and the probability that the neighbor nodes of *v*_*j*_ are labeled with the class *L*_*c*_ is greater. Considering all the neighbor nodes of *v*_*i*_, the probability that the node *v*_*i*_ has the class *L*_*c*_ is proportional to the sum of the class propagating distributions of all the neighbor nodes. After normalization, *P*_*ic*_ can be described by

(5) $$\begin{array}{c} P_{ic}=p\left(y_{i}=c\;|\;\delta_{i}\right)=\frac{\sum_{j=1}^{N}q_{jc}W_{ij}}{\sum_{k=1}^{N}W_{ik}}. \end{array}$$

The classes of unlabeled nodes are calculated in an iterative manner. At the beginning, all class distributions of unlabeled nodes are initialized with the vectors in which all elements are equal to a constant 1/*M*, since the classes of these nodes are unknown. Then, the class propagating distributions of all nodes are calculated using (4). The class distributions of unlabeled nodes are then calculated using (5). Repeat the above two steps while the class distributions are not stable. When the class distributions are stable, the final class distributions of unlabeled nodes are obtained. The class label, to which the maximal element in the class distribution of the node *v*_*i*_ corresponds, is assigned to the node *v*_*i*_. 

The class distributions and the class propagating distributions are written in the matrix form, denoted by **P** and **Q**,

(6) $$\begin{array}{c} P={\left[P_{1}^{T},\ldots ,P_{i}^{T},\ldots ,P_{N}^{T}\right]}^{T},\\ Q={\left[q_{1}^{T},\ldots ,q_{i}^{T},\ldots ,q_{N}^{T}\right]}^{T}. \end{array}$$

Equations (4) and (5) are written in matrix form

(7) $$\begin{array}{c} Q=\text{nrow}\left(W\right)P,\\ P=\text{nrow}\left(W\right)Q, \end{array}$$

where the function nrow() is the normalization for each row. The classification algorithm is displayed in Algorithm 1, where *y*_*i*_ denotes the classes of the node *v*_*i*_. Let the matrix **Y** denote {*y*_*i*_}_*i*=1_^*N*^. The matrix **Y** has some unknown elements, which are the classes of the unlabeled nodes. This algorithm calculates these elements. For convenience, the algorithm in Algorithm 1 is called CPD for short.

CPD algorithm is similar to MRW and wvRN, which calculate the classes of unlabeled nodes in an iterative manner. MRW and wvRN calculate the class of a node based on the classes of its neighbor nodes. CPD calculates the node class using a probabilistic approach, instead of the classes of neighbor nodes.

## 4. Experiments and Results

### 4.1. Experiment Setup

Six real networks are used to examine the performance of the proposed CPD algorithm, including Citeseer, Cora, and four networks in WebKB dataset [11]. WebKB dataset has four networks, including Texas, Cornell, Wisconsin, and Washington. In these networks, nodes are web pages of the four universities and edges are hyperlinks between them. Citeseer and Cora are paper citation networks built with citation relationship. The information of the experimental data is listed in Table 1. 

In the experiments, micro*-F1* and macro*-F1* are used as the evaluation metrics. micro*-F1* and macro*-F1* are real numbers between 0 and 1; the larger they are the better the classification algorithms are. We calculate micro*-F1* and macro*-F1* with (8) and (9) used in [9]. *t*_*ic*_ indicates the true class label in original datasets while *y*_*ic*_ indicates the class label returned by classification algorithms. If the true class label of the node *v*_*i*_ is *L*_*c*_, *t*_*ic*_ is equal to 1, otherwise *t*_*ic*_ = 0. If the *v*_*i*_'s label returned by classification algorithms is *L*_*c*_, *y*_*ic*_ is equal to 1, otherwise *y*_*ic*_ = 0.

(8) $$\begin{array}{c} \text{micro-}F1=2\frac{\sum_{i,c}\left(y_{ic}t_{ic}\right)}{\sum_{i,c}\left(y_{ic}+t_{ic}\right)}, \end{array}$$

(9) $$\begin{array}{c} \text{macro-}F1=\frac{2}{M}\sum_{c=1}^{M}\frac{\sum_{i,c}\left(y_{ic}t_{ic}\right)}{\sum_{i,c}\left(y_{ic}+t_{ic}\right)}. \end{array}$$

### 4.2. Experiment Results

#### 4.2.1. Classification Performance

In order to test the classification performance of the proposed CPD algorithm, CPD is compared with four methods on real networks. The four baseline methods are BLC, wvRN, MRW, and SocioDim. BLC is a collective inference method based on Bayesian local classifier. wvRN is the collective inference method without training classifiers. MRW is a classification method based on random walk. SocioDim is a classification method based on extracting latent attributes of nodes. CPD, wvRN, and MRW calculate the node classes in an iterative manner. In the realization of them, the termination condition of iterations is *ε* = 10^−5^/*N* and the maximal iteration number is 500. 

**Figure 1:**
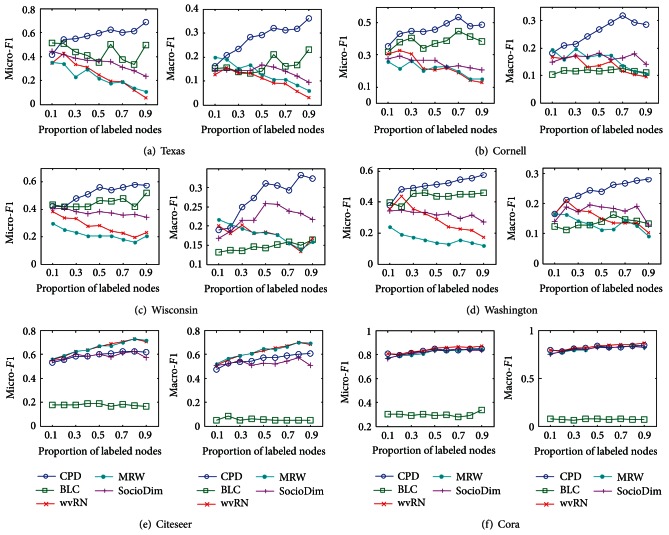
Results of comparison experiments.

Let *r* denote the proportion of the labeled nodes in all nodes of a network. According to the value of *r*, the labeled nodes of a network are picked out randomly. The rest nodes are used as the test data, whose classes are calculated by classification methods. In this way, the labeled nodes of a network are produced for 10 times at a value of *r*. The average micro-*F1* and macro-*F1* are plotted in Figure 1.

Figure 1 displays that the performance of CPD is better than that of the other four methods on the first four networks. On Citeseer and Cora networks, CPD underperforms the other four methods. These experimental results can be explained by the homophily degree of these networks. According to [11], the homophily degree of a network can be indicated by the average percentage by which a node's neighbor is of the same class label. Consequently, the homophily degree of a network can be calculated using

(10) $$\begin{array}{c} \text{homophily}=\frac{\sum_{i=1}^{N}\left(s_{i}/d_{i}\right)}{N}, \end{array}$$

where *d*_*i*_ denotes the number of nodes that connect to the node *v*_*i*_ and *s*_*i*_ denotes the number of nodes that connect to the node *v*_*i*_ and have the same class with *v*_*i*_. The homophily degrees of the networks in Table 1 are calculated and the results are listed in Table 2. The homophily degrees of first four networks are very low, so they are the networks with heterophily. 

MRW and wvRN are homophily-based methods, which calculate the classes of unlabeled nodes using the classes of their neighbor nodes, so they perform better on the Citeseer network and the Cora network, which are both of high homophily. The first four networks are of heterophily, where most of connected nodes have different classes, so the homophily-based methods performance declines. BLC, SocioDim, and CPD abandon the homophily assumption, so they achieve better performance than MRW and wvRN. These experiments show that CPD has better performance on the networks with heterophily.

#### 4.2.2. Convergence

**Figure 2:**
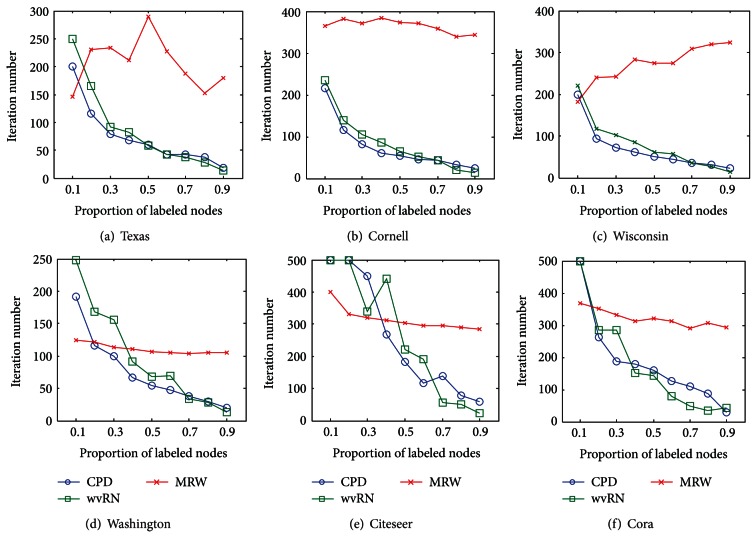
The comparison of iteration number.

CPD calculates class labels of nodes in the iterative manner and 500 iterations are used in the above experiments. The issue that concerns us is whether CPD is able to converge within 500 iterations. In this subsection, the convergence of CPD is studied through experiments. We use *ε* = 10^−5^/*N* as the termination condition of iterations, and the maximum iteration number is 500. The iteration numbers when CPD terminates are plotted in Figure 2. 

Because MRW and wvRN require iterative calculation, their iteration numbers are also plotted in Figure 2 for comparison. Figure 2 shows that CPD can satisfy the termination condition of iterations on the first four networks and its iteration number is less than those of wvRN and MRW. It means that CPD is convergent on the networks with heterophily. 

## 5. Conclusions

Many classification methods in networked data classify nodes based on homophily assumption using their neighbor nodes. In real world, there are many networks with heterophily, in which the classes of unlabeled nodes are hardly calculated using their neighbor nodes. This paper focuses on such problem to develop a novel approach, which utilizes a probabilistic approach to measure the class influence between two connected nodes. The experiments on real datasets show that the proposed method has better performance on the networks with heterophily.

## Figures and Tables

**Algorithm 1:**
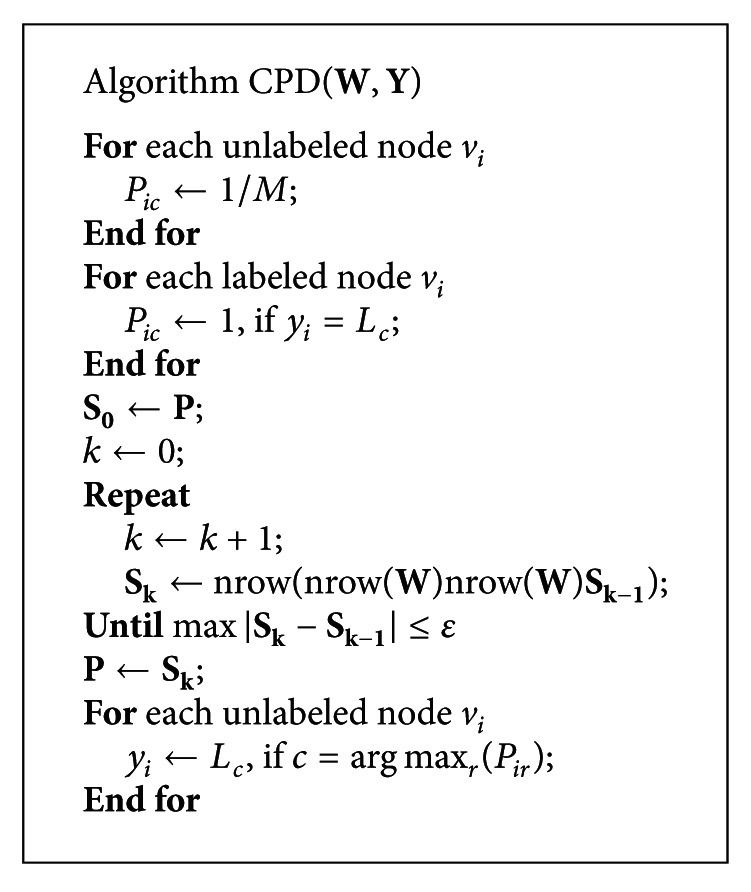
The classification algorithm based on class propagating distributions.

**Table 1:** The information about experimental data.

Name	Number of nodes	Number of edges
Texas	187	578
Cornell	195	569
Wisconsin	265	938
Washington	230	783
Citeseer	3312	4598
Cora	2708	10556

**Table 2:** The homophily degrees of the networks in Table 1.

Name	Homophily degree
Texas	0.1068
Cornell	0.1212
Wisconsin	0.1868
Washington	0.2698
Citeseer	0.7256
Cora	0.8252
